# Regulation of the Nrf2/HO-1 pathway in chronic obstructive pulmonary disease-induced muscle atrophy using Jinshui Liujian decoction and Bajitian pills: insights from network pharmacology and animal models

**DOI:** 10.1186/s41065-025-00432-5

**Published:** 2025-04-21

**Authors:** Bai-Yang Lin, Li Bai, Sheng-Long Wang

**Affiliations:** 1 Department of Respiratory, Shanxi Provincial People’s Hospital, Taiyuan, 030001 China; 2 Department of Respiratory, Shanxi Provincial Integrated TCM and WM Hospital, The Third Clinical College of Shanxi University of Chinese Medicine, NO.13 of Fudong Road, Xinghualing District, Taiyuan, 030001 China

**Keywords:** Bajitian pills, Chronic obstructive pulmonary disease, Jinshui Liujian decoction, Muscle atrophy, Nrf2/HO-1 pathway

## Abstract

**Objective:**

This study aimed to investigate the therapeutic effects of the combination of modified Jinshui Liujian decoction and Bajitian pills (TCM) on chronic obstructive pulmonary disease (COPD)-induced muscle atrophy using network pharmacology and animal model experiments.

**Methods:**

Network pharmacology technique has been employed to analyze the potential targets of TCM on treating COPD. In vivo, COPD mice model was induced by lipopolysaccharide (LPS) combined with smoke treatment. To comparing the protective effects of TCM on this disease, these parameters including general condition, serum inflammatory factors, protein expression levels, gene copies, and histopathological changes in the lungs and gastrocnemius muscle mass have been further assessed in mouse in different groups.

**Results:**

Network pharmacology analysis identified 203 intersecting targets, primarily associated with apoptosis, phosphorylation, and inflammatory response. Animal experimental demonstrated that TCM could significantly improve the decreased skeletal muscle mass (*p* < 0.001), abnormal histopathologic morphology, decreased superoxide dismutase (SOD, *p* < 0.05), increased levels of serum malondialdehyde (MDA, *p* < 0.001) and tumor necrosis factor-α (TNF-α, *p* < 0.001) as compared to model group. Further mechanism exploration showed that a significant increase on the gene and proteins levels of nuclear factor erythroid 2-related factor 2 (NRF2, *p* < 0.05) and heme oxygenase-1 (HO-1, *p* < 0.05) have been observed in TCM-treated animals compared with that of in model animals. Interestingly, some indicators (serum MDA/SOD/TNF-α, RNA and protein levels of NRF2 and HO-1) showed more positive changes in TCM combined with western medicine (TCM-WN) - treated animals compared with that of TCM-treated animals.

**Conclusion:**

Modified Jinshui Liujian decoction and Bajitian pills effectively mitigate muscle atrophy associated with COPD by modulating the Nrf2/HO-1 pathway through multi-target mechanisms. The combined TCM and WM therapy demonstrates enhanced therapeutic efficacy compared to monotherapy.

**Supplementary Information:**

The online version contains supplementary material available at 10.1186/s41065-025-00432-5.

## Introduction

Chronic obstructive pulmonary disease (COPD) is one of the most common respiratory disorders worldwide and the third leading cause of fatality on a global scale [1]. This disease demonstrates an age-dependent epidemiological pattern, with prevalence rates escalating significantly beyond the sixth decade of life. The epidemiological data estimate a global prevalence of 10.3% among individuals aged ≥ 60 years [2]. COPD is primarily characterized by airflow obstruction, with systemic effects affecting multiple organ systems. The complex pathophysiology of COPD involves various pathways, for example, chronic systemic inflammation, oxidative stress amplification, and protease-antiprotease homeostasis disruption. These synergistic pathological processes induce structural degradation of pulmonary tissues through alveolar wall destruction and small airway remodeling, ultimately culminating in progressive and irreversible pulmonary impairment [3]. Up to 50% of patients with COPD experience malnutrition and muscle atrophy, conditions that reduce quality of life, worsen prognosis, and complicate treatment [4]. Identifying therapeutic interventions is crucial for improving disease outcomes. Integrative approaches combining traditional Chinese and Western medicine have evolved, with the modified Jinshui Liujian Decoction widely used in COPD treatment, demonstrating efficacy in reducing systemic inflammation [5]. Bajitian pills, described in the ‘Taiping Shenghui Fang,’ are known for their kidney-tonifying, muscle-strengthening, and anti-inflammatory properties. The combination and modification of these two formulations aim to relieve cough, reduce phlegm, attenuate inflammation and oxidative stress, and enhance muscle strength and mass.

Network pharmacology is a powerful tool for elucidating the mechanisms underlying traditional Chinese medicine (TCM) interventions, providing reliable evaluations of TCM pharmacology based on molecular structures. This approach emphasizes the multi-target and multi-pathway effects of the formulations [6]. Screening and research have identified that activation of the nuclear factor erythroid 2-related factor 2 (Nrf2)/antioxidant response element (ARE) pathway is crucial for maintaining redox homeostasis and protecting skeletal muscle; this pathway may serve as a potential therapeutic target for muscle atrophy [7]. *Nrf2* is a cellular protective gene that forms a defense pathway against inflammation and oxidative stress [8]. This study employs network pharmacology and animal models to investigate how the modified Jinshui Liujian decoction combined with Bajitian pills modulates the Nrf2/Heme oxygenase-1 (HO-1) pathway to mitigate muscle atrophy in a mouse model of COPD.

## Materials and methods

### Network pharmacology analysis

#### Target identification for Jinshui Liujian decoction combination with Bajitian pills

The chemical constituents of the herbs in the compound prescription, including *Chenpi*, *Qingbanxia*, *Fuling*, *Gancao*, *Danggui*, *Shudi*, *Xingren*, *Jiegeng*, *Zhebeimu*, *Ziyuan*, *Kuan-donghua*, *Suzi*, *Bajitian*, *Tusizi*, *Wuweizi*, *Shanyurou*, *Buguzhi*, and *Niuxi*, were identified through the TCMSP and TCMIP databases. Active components were selected based on the criteria of oral bioavailability (OB) OB ≥ 30% and drug-likeness (DL) ≥ 0.18. For compounds lacking recorded targets in the TCMSP database, canonical SMILES notations were retrieved from the PubChem database and entered into the Swiss Target Prediction platform. Target prediction was conducted for *Homo sapiens* to identify the relevant drug targets. Data files were downloaded, and targets with a probability of zero (*p* = 0) as well as duplicates were removed. Gene names for the targets were obtained from the UniProt database, and a de-duplication analysis was conducted to finalize the target dataset.

#### Target retrieval for sarcopenia and common target screening

Targets associated with sarcopenia, muscle atrophy, and muscle loss were obtained from the GeneCards database by using relevant keywords. The results were filtered based on a relevance score of ≥ 10, with the median score selected for further analysis. Additional disease-related targets were obtained from the OMIM and DisGeNet databases. Duplicate entries were removed, and the resulting targets were subjected to visualization analysis to facilitate further evaluation.

#### Protein-protein interaction (PPI) network construction and potential target screening

The disease-related targets for sarcopenia and drug-related targets from the compound prescription were entered into the MicroBio Informatics platform to identify intersections. The intersecting targets were then imported into the STRING database to construct a PPI network, with the species set as *Homo sapiens* and a confidence threshold of medium confidence (> 0.700). Default parameters were applied for all other settings. TSV files generated from STRING were subsequently uploaded into Cytoscape 3.9.1 for topological analysis via the ‘Analyze Network’ function. Targets were filtered based on a degree threshold of ≥ 18.59, leading to the identification of potential therapeutic targets.

#### Gene ontology (GO) and kyoto encyclopedia of genes and genomes (KEGG) enrichment analysis

Intersecting targets were subject to GO and KEGG enrichment analyses using the DAVID database. GO biological function analysis was divided into three categories: Biological Process (BP), Cellular Component (CC), and Molecular Function (MF). Data within each category were filtered with a significance threshold of *p* < 0.01, and the results were visualized as bar charts using the MicroBio Informatics platform. For KEGG enrichment analysis, pathways were filtered using a threshold of *p* < 0.01, and the top 30 pathways were visualized using a bubble chart.

### Experimental verification

#### Animals

Male BALB/c mice aged 6 to 8 weeks and weighing 18 to 22 g, were obtained from Beijing Huafukang Biological Technology Co., Ltd., with production license SCXK (Jing) 2019-0008. The mice were housed in a controlled environment with a room temperature of 20 ℃ to 26 ℃ and relative humidity ranging from 40 to 70%.

#### Instruments and equipment

The Jinshui Liujian decoction combined with Bajitian pills (formulation: *Chenpi* 10 g, *Qingbanxia* 10 g, *Fuling* 16 g, *Gancao* 6 g, *Danggui* 12 g, *Shudi* 10 g, *Xingren* 10 g, *Jiegeng* 10 g, *Zhebeimu* 10 g, *Ziyuan* 10 g, *Kuan-donghua* 10 g, *Suzi* 9 g, *Bajitian* 10 g, *Tusizi* 10 g, *Wuweizi* 10 g, *Shanyurou* 10 g, *Buguzhi* 10 g, *Niuxi* 10 g); budesonide inhalation suspension (Changfeng Pharmaceutical Co., Ltd.); malondialdehyde (MDA) and superoxide dismutase (SOD) assay kits (WST-1 method); hematoxylin-eosin (HE) staining kit (Nanjing Jiancheng Bioengineering Institute); mouse tumor necrosis factor-alpha (TNF-α) assay (Shanghai Kexing); Nrf2 and HO-1 primary antibodies (Proteintech); cDNA synthesis and PCR detection kits (Shanghai Bioengineering Co., Ltd.); microplate reader (Beijing Pulang Technology Co., Ltd.); automated chemiluminescence, fluorescence, and gel imaging system (Beijing Saizhi); real-time quantitative PCR instrument (ThermoFisher Scientific).

#### Animal grouping, modeling, and drug administration

BALB/c mice were assigned randomly to control, COPD model, Western medicine (WM), TCM, and TCM-WM combination groups. Mice in the drug administration groups were placed in an organic glass smoke chamber and exposed to the smoke from 12 cigarettes twice daily, with each session lasting 30 min over 60 consecutive days. On days 1 and 15, under 10% chloral hydrate anesthesia, 100 µL of lipopolysaccharide (1 mg/mL) was administered intratracheally to establish the COPD model, with no smoking on the modeling days. The control group was placed in the chamber with normal ventilation, and 0.9% sodium chloride was administered intratracheally on days 1 and 15.

On day 28 of the modeling, the TCM group was administered the modified Jinshui Liujian decoction in combination with Bajitian pills by gavage one hour before smoking, once daily, and underwent aerosol inhalation of sterile injection water once daily. The WM group received budesonide (1 mg/day) via inhalation, along with 0.9% sodium chloride solution by gavage. The TCM-WM combination group received both TCM by gavage and aerosol therapy. The model and control groups were treated with sterile injection water via aerosol inhalation and gavage of 0.9% sodium chloride solution for 21 consecutive days.

#### Measurement of serum inflammation and oxidative stress markers

Twenty-four hours after the last administration, 1 mL of blood was drawn from the apex of the heart, centrifuged within 1 h to obtain the serum (3000 rpm, 10 min), and serum samples were analyzed for MDA, SOD, and TNF-α levels according to the assay kit instructions.

#### Detection of lung and muscle pathological changes

Following blood collection from the apex of the heart, the mice were euthanized, and the chest cavity was opened to harvest the lungs. The gastrocnemius muscles from both hind limbs were weighed. The right gastrocnemius muscle and the middle and lower lobes of the right lung were fixed in 4% paraformaldehyde, embedded in paraffin, and sectioned. The tissue sections were stained using the HE staining kit, and the lung tissue structure and skeletal muscle fiber morphology were examined under a microscope.

#### Detection of Nrf2 and HO-1 protein expression in skeletal muscle

Skeletal muscle was homogenized to extract proteins to measure the expression of Nrf2 and HO-1 proteins using the BCA protein assay kit (following the manufacturer’s instructions). Protein samples were loaded onto 10% separating and 4% stacking gels, and electrophoresis was run at 80 V. Wet transfer was conducted at 250 mA for 80 min. Primary antibodies (Nrf2 at 1:5000 dilution, HO-1 at 1:3000, β-tubulin at 1:2000) were incubated with gentle shaking at 4 °C overnight. After washing, HRP-labeled secondary antibodies (1:5000) were incubated at room temperature for 60 min. Films were scanned in a darkroom, and ImageJ software was used to analyze the gray values of each group’s films.

#### Quantification of Nrf2 mRNA in skeletal muscle by PCR

Thirty milligrams of skeletal muscle were homogenized to extract and precipitate RNA. The RNA precipitate was dissolved in 20 to 50 µL DEPC-treated water. One microliter of the solution was diluted 1:100 and measured at OD260 to determine the concentration. Real-time quantitative PCR kits amplified cDNA samples and standards for the *Nrf2* and *HO-1* genes. The primer sequences were:

Nrf2 forward: 5’-CTGGCTGATACTACCGCTGTTC-3’,

reverse: 5’-GTGGAGAGGATGCTGCTGAAAG-3’;

HO-1 forward: 5’-CAGAAGAGGCTAAGACCGCC-3’,

reverse: 5’-CTCTGACGAAGTGACGCCAT-3’.

The cycle threshold value was derived from the amplification curve, and the copy number was determined.

#### Statistical analysis

Statistical analysis was performed using SPSS 22.0 software. Experimental data are presented as mean ± standard deviation (SD). One-way analysis of variance (ANOVA) was used for group comparisons. If variance homogeneity was confirmed, the LSD-t test was used for pairwise comparisons; if not, the Games–Howell test was applied. A *p* < 0.05 was considered statistically significant.

## Results

### Screening of active components in Jinshui Liujian decoction and Bajitian pill combination

By collating target data from the Swiss Target Prediction, TCMSP, and TCMIP databases, a preliminary screening revealed 244 active components, distributed as follows: *Chenpi* (5), *Qingbanxia* (12), *Fuling* (15), *Gancao* (52), *Danggui* (2), *Shudi* (2), *Xingren* (17), *Jiegeng* (7), *Zhebeimu* (7), *Ziyuan* (16), *Kuandonghua* (15), *Suzi* (16), *Bajitian* (18), *Tusizi* (10), *Wuweizi* (8), *Shanyurou* (14), *Buguzhi* (16), and *Niuxi* (18). These were associated with 813 identified action targets.

### Prediction of targets for Jinshui Liujian decoction and Bajitian pill combination in the intervention of muscle atrophy

Following de-duplication across three disease-related databases, 1,643 disease targets were identified. The intersection between these disease-related targets and the compound’s action targets was conducted using the MicroBio Informatics platform, yielding 203 common targets.

### Construction of the PPI network and screening of potential targets

**Fig. 1:**
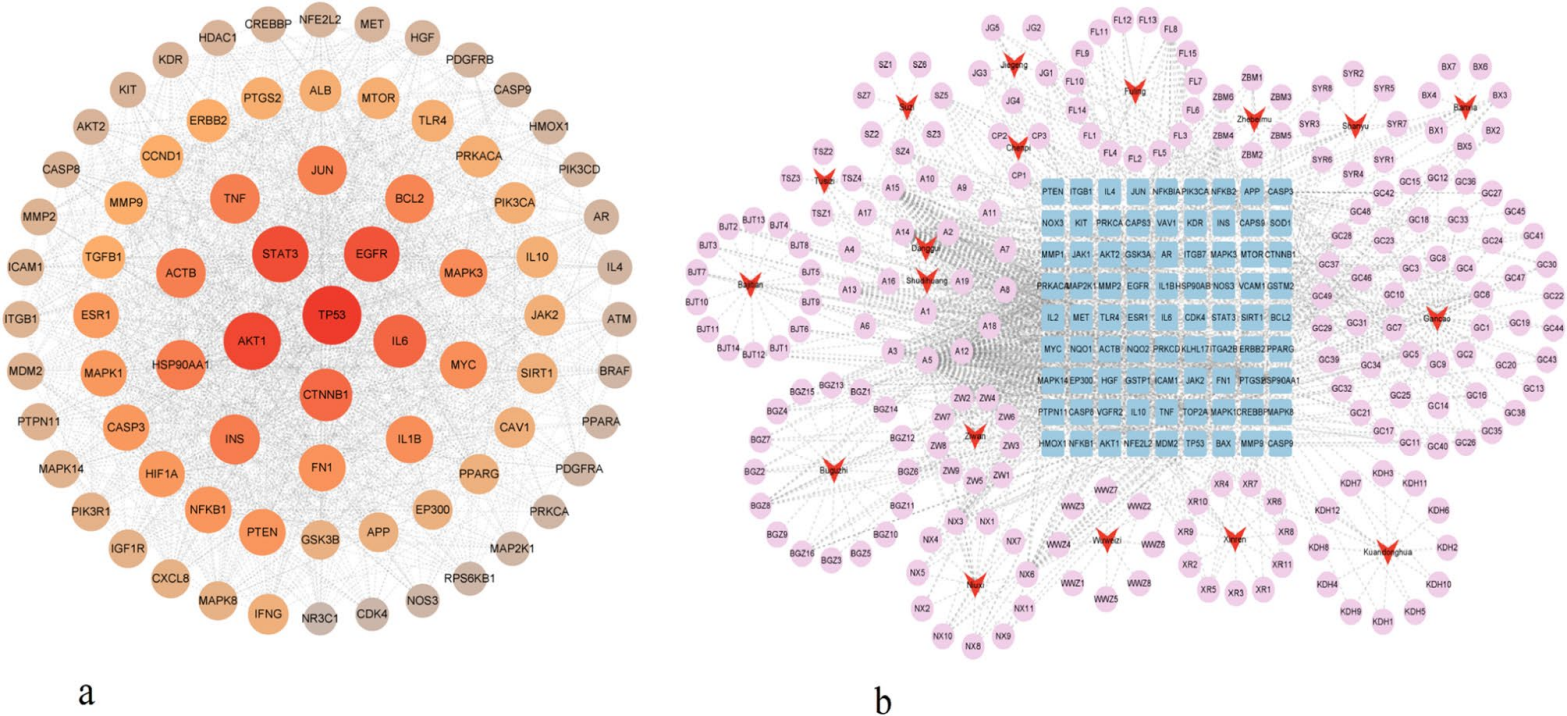
(**a**) Visualization of the PPI network for core targets of the modified Jinshui Liujian decoction and Bajitian pill combination in treating sarcopenia. Note: The size and color intensity of the circles correspond to the degree values; larger and darker circles signify higher degree values. (**b**) Drug-component-target network diagram

PPI network analysis identified 193 nodes, including 10 isolated targets, and 1,794 edges. The size and color of the nodes represented degree values, with larger and darker nodes indicating stronger connectivity within the network. Using the screening condition of “Degree unDir ≥ 18.59,” 74 core targets were identified, as shown in Fig. 1a. Key interacting proteins in the PPI network included TP53, STAT3, AKT2, TNF, IL-6, JUN, and HMOX1. The relevant target information was then categorized, and a drug-component-disease target network was constructed, as demonstrated in Fig. 1b.

### GO functional enrichment analysis for Jinshui Liujian decoction and Bajitian pill combination in the intervention of muscle atrophy

**Fig. 2:**
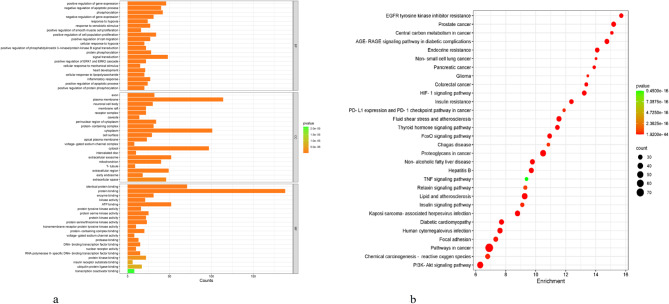
(**a**) Bar chart of GO enrichment analysis for intersection target proteins. (**b**) KEGG pathway enrichment analysis diagram. Note: The bubble color represents the log-transformed P-values, while the bubble size reflects the number of genes enriched in each pathway

GO functional enrichment analysis using the DAVID data platform identified 485 significant GO terms (*p* < 0.01), including 314 terms under BP, 78 under CC, and 93 under MF. A summary of the GO functional enrichment analysis is presented in a bar chart in Fig. 2a. Top-ranked BP terms included positive regulation of gene expression, negative regulation of apoptotic process, phosphorylation, and inflammatory response. The top-ranked CC terms included axon, plasma membrane, neuronal cell body, and membrane raft. The leading MF terms included identical protein binding, transmembrane receptor protein tyrosine kinase activity, and protein serine/threonine kinase activity.

### KEGG pathway enrichment analysis

KEGG pathway enrichment analysis using the DAVID platform identified 167 significant signaling pathways (*p* < 0.01). A bubble chart of the top KEGG pathways is displayed in Fig. 2b. The most enriched pathways included those associated with cancer, proteoglycans in cancer, TNF signaling, PI3K-Akt signaling, and resistance to EGFR tyrosine kinase inhibitors.

### Experimental validation of results

#### Comparative application of baseline characteristics among the five groups of mice

Before COPD model induction, all five groups showed normal neurological function and consistent food consumption, with no significant differences in body mass (*p* > 0.05). Following COPD model establishment (including the COPD model, WM, TCM, and TCM-WM combination groups), a notable decline in alimentary intake, diminished physical activity, dull fur, and reduced body mass were observed across the groups, with the mean body mass in the model groups being significantly lower than that of the control group.

However, post-intervention analysis showed a significant increase in body mass among the three therapeutic groups compared to the model group (*p* < 0.05), with the TCM-WM combination group showing the largest increase (*p* < 0.001), as detailed in Table 1. Compared to the control group, a marked decrease in skeletal muscle mass was recorded in the model groups (*p* < 0.001). The TCM group showed a significant increase in gastrocnemius muscle mass compared to the model group (*p* < 0.001), with the TCM-WM combination and WM groups exhibiting even larger increases compared to the TCM group, as detailed in Table 2.

**Table 1:** Comparative analysis of body mass before and after modeling in five groups of mice (g, mean ± SD), COPD model, Western medicine (WM), traditional Chinese medicine (TCM), and TCM-WM combination groups

Group	*n*	Before Modeling(g)	After Modeling(g)
Control	8	23.21 ± 0.54	26.18 ± 0.33
COPD model	8	22.93 ± 0.58	18.92 ± 0.30
TCM	8	22.63 ± 0.75^*^	24.01 ± 0.29^*^
WM	8	22.70 ± 0.64^#^	24.20 ± 0.37^#^
TCM-WM combination^@^	8	22.89 ± 0.63^@^	24.94 ± 0.39^@^
f value		1.12	541.90
P value		0.36	< 0.001

**Note**: Compared with the control group, *P* < 0.001 for the four model groups; compared with the model group, *P* < 0.001 for the other three groups; compared with the WM group (^#^), ^*^P = 0.27,^@^*P* < 0.001

**Table 2:** Comparative analysis of skeletal muscle mass among five groups of mice (g, mean ± SD)

Group	*n*	Skeletal Muscle Mass(g)
Control	8	0.308 ± 0.011
COPD model	8	0.175 ± 0.012
TCM	8	0.241 ± 0.008^*^
WM	8	0.263 ± 0.009^#^
TCM-WM combination	8	0.265 ± 0.014^@^
f value		156.49
P value		< 0.001

**Note**: Compared with the control group, *P* < 0.001 for the four model groups; compared with the model group, *P* < 0.001 for the other three groups; compared with the WM group (^#^), ^*^P < 0.001,^@^*P* = 0.685

#### Comparative analysis of pulmonary and skeletal muscle histopathological changes across the five experimental groups

**Fig. 3:**
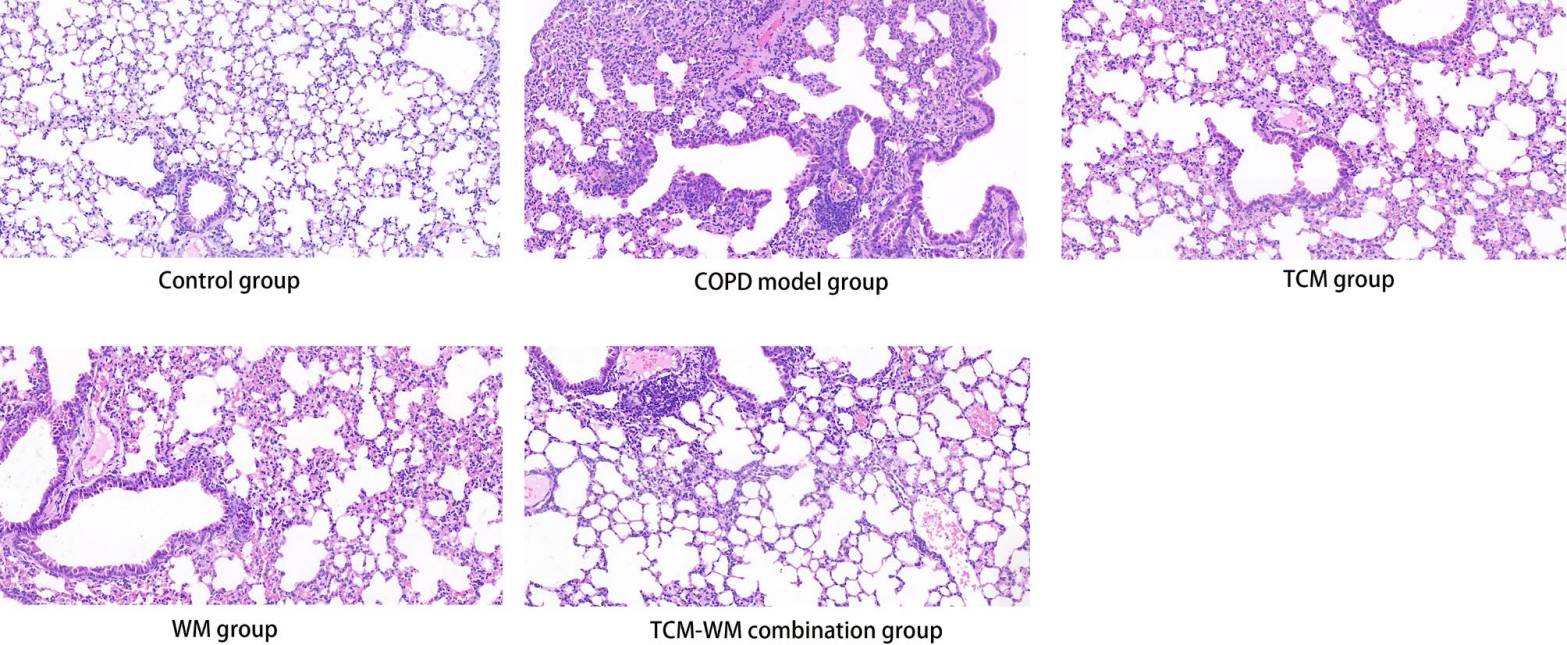
HE staining of lung tissues in the five mice groups

Histological examination of pulmonary tissues through HE staining for the different groups is presented in Fig. 3. The control group showed structurally intact bronchial and alveolar walls. In contrast, the model group showed pronounced bronchial wall thickening, disrupted alveolar architecture, partial rupture of alveolar walls, and extensive infiltration of inflammatory cells accompanied by enlarged alveolar spaces. The WM, TCM, and TCM-WM combination groups displayed remaining thickening of bronchial and alveolar walls with partial alveolar wall rupture; however, these pathological changes were less pronounced compared to the model group, with the TCM-WM combination group showing the most significant histological improvement.

**Fig. 4:**
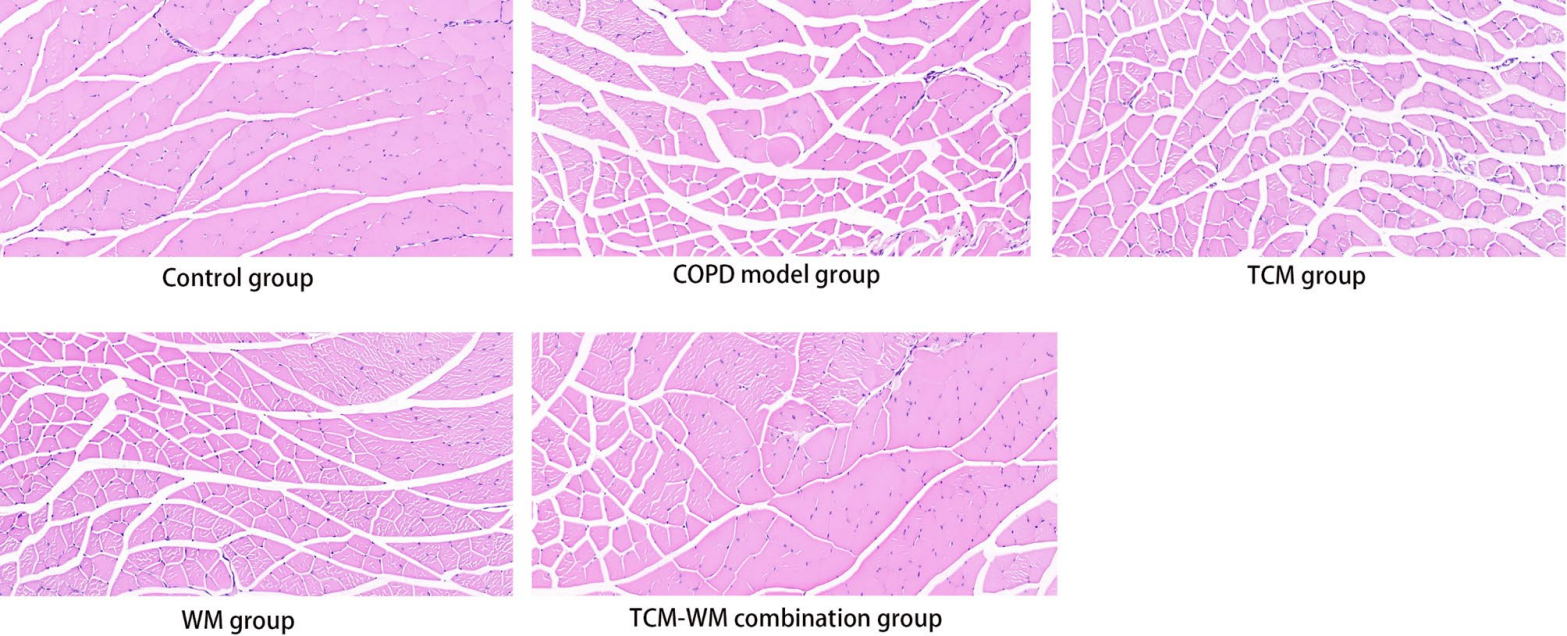
HE staining of skeletal muscles in the five mice groups

Regarding skeletal muscle morphology, the control group showed orderly aligned muscle fibers with minimal interstitial spacing, as demonstrated in Fig. 4. The model group showed atrophied gastrocnemius muscles, characterized by sparse and disordered fiber alignment, widened interstitial spaces, and a reduction in type I muscle fibers, consistent with the pathophysiological changes observed in COPD-associated sarcopenia [9]. The WM, TCM, and TCM-WM combination groups showed relatively narrower interstitial spaces and more orderly fiber alignment compared to the model group, with the TCM-WM combination group showing morphology most similar to that of the control group.

#### Comparative analysis of serum inflammatory and oxidative stress biomarkers across the five experimental groups

Compared to the control group, the model groups showed elevated serum levels of MDA and TNF-α, along with reduced SOD levels. When compared to the model group, the TCM, WM, and TCM-WM combination groups showed significantly lower serum MDA and TNF-α levels, along with elevated SOD concentrations (*p* < 0.05), as detailed in Table 3. No significant differences in serum MDA and SOD levels were observed between the TCM and WM groups. However, the TCM-WM combination group showed a more significant reduction in MDA and TNF-α levels and a greater increase in SOD levels, relative to both the TCM and WM groups (*p* < 0.05), with MDA concentrations similar to those in the control group. These results suggest that the pharmacological interventions effectively modulated serum inflammatory and oxidative stress markers, with the TCM-WM combination therapy demonstrating the most efficacious results.

**Table 3:** Comparative analysis of serum MDA, SOD, and TNF-α levels in five groups of mice

Group	*n*	MDA(nmol/mL)	SOD(U/mL)	TNF-α(ng/L)
Control	8	10.13 ± 0.98	65.73 ± 3.39	436.58 ± 9.79
COPD model	8	19.40 ± 1.38	53.42 ± 2.80	514.50 ± 15.31
TCM	8	13.56 ± 1.81^*^	58.61 ± 2.31^*^	464.76 ± 14.60^*^
WM	8	14.08 ± 1.59^#^	56.85 ± 2.35^#^	480.42 ± 10.18^#^
TCM-WM combination	8	11.15 ± 1.65^@^	61.01 ± 3.68^@^	459.24 ± 13.03^@^
f value		45.54	19.45	40.908
P value		< 0.001	< 0.001	< 0.001

**Note**: For MDA levels, compared with the control group, @*P* = 0.183, *P* < 0.001 for the other three groups; compared with the model group, *P* < 0.001 for the other three groups; compared with the WM group (^#^), ^*^P = 0.502,^@^*P* = 0.003

For SOD levels, compared with the control group, *P* < 0.05 for all four model groups; compared with the model group, *P* < 0.05 for the other three groups; compared with the WM group (^#^), ^*^P = 0.242,^@^*P* = 0.008

For TNF-α levels, compared with the control group, *P* < 0.001 for all four model groups; compared with the model group, *P* < 0.001 for the other three groups; compared with the WM group (^#^),^*^P = 0.019,^@^*P* = 0.002

#### Comparative analysis of Nrf2 and HO-1 protein expression in skeletal muscle across the five experimental groups

**Fig. 5:**
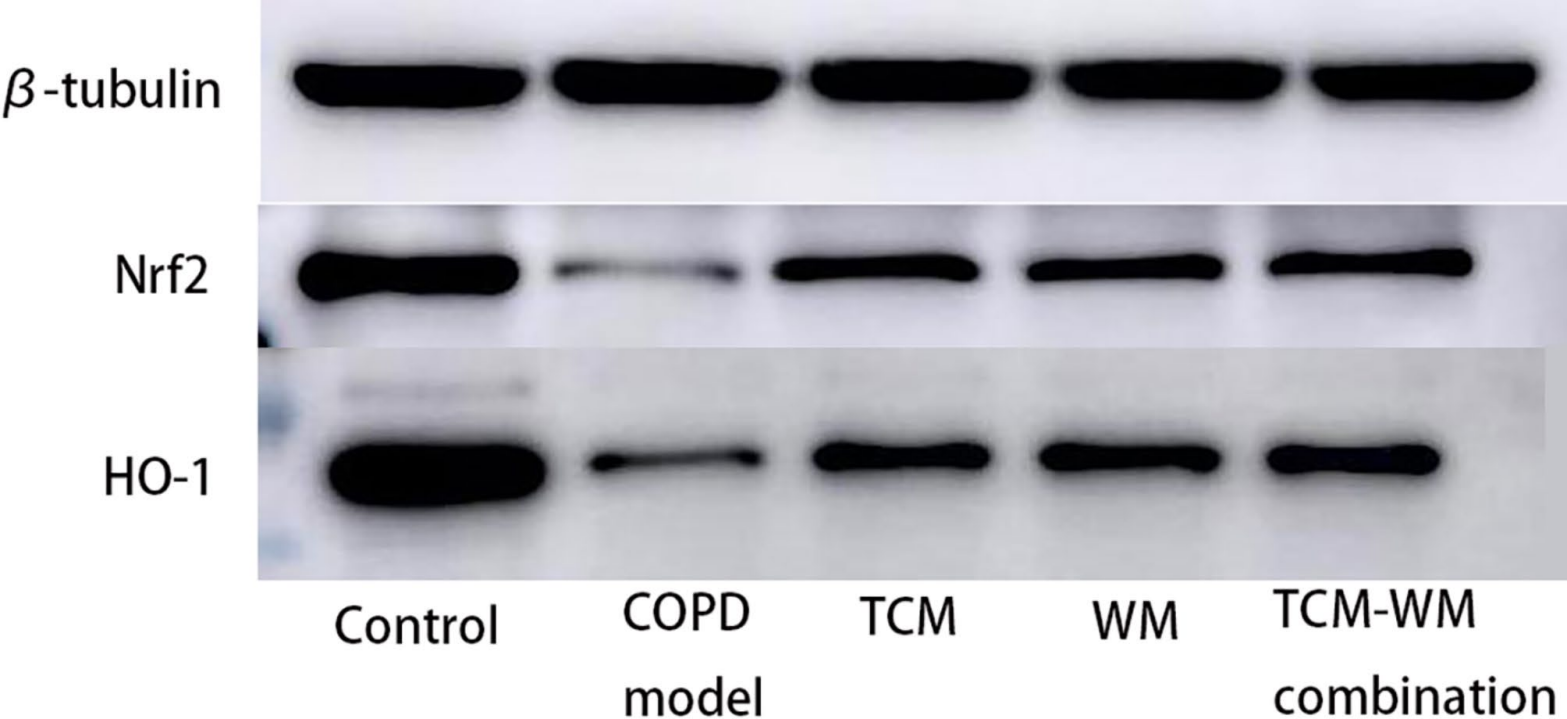
Expression of Nrf2 and HO-1 proteins in skeletal muscles of different groups of mice

The model group showed a significant decrease in Nrf2 and HO-1 protein expression levels within skeletal muscle (*p* < 0.05). In contrast, the TCM, WM, and TCM-WM combination groups displayed elevated levels of Nrf2 and HO-1 protein expression compared to the model group (*p* < 0.05), as detailed in Table 4; Fig. 5. Nrf2 expression was similar between the WM and TCM groups, with no significant difference. However, Nrf2 expression in the TCM-WM combination group was higher than both the TCM and WM groups (*p* < 0.05), though it remained lower than in the control group. HO-1 expression was highest in the TCM-WM combination group, but still lower than in the control group, with significant differences between groups (*p* < 0.05). These findings suggest that both TCM and TCM-WM combination therapies can enhance Nrf2 and HO-1 expression within skeletal muscle, with the TCM-WM combination therapy yielding superior outcomes.

**Table 4:** Comparative analysis of Nrf2 and HO-1 expression levels in skeletal muscles of five groups of mice

Group	*n*	Nrf2	HO-1
Control	8	1.000 ± 0.000	1.000 ± 0.000
COPD model	8	0.294 ± 0.355	0.395 ± 0.092
TCM	8	0.661 ± 0.053^*^	0.562 ± 0.103^*^
WM	8	0.637 ± 0.080^#^	0.735 ± 0.141^#^
TCM-WM combination	8	0.727 ± 0.081^@^	0.860 ± 0.146^@^
f value		147.098	37.895
P value		< 0.001	< 0.001

For Nrf2 expression levels, compared with the control group, *P* < 0.001 for all four model groups; compared with the model group, *P* < 0.001 for the other three groups; compared with the WM group (^#^), ^*^P = 0.403,^@^*P* = 0.004

For HO-1 expression levels, compared with the control group, *P* < 0.05 for all four model groups; compared with the model group, *P* < 0.05 for the other three groups; compared with the WM group (^#^), ^*^P = 0.003, ^@^*P* = 0.029

#### Comparative analysis of Nrf2 and HO-1 gene copy numbers in skeletal muscle across the five experimental groups

Real-time quantitative PCR assays showed a decrease in *Nrf2* and *HO-1* gene expression within the skeletal muscle of the model group (*p* < 0.05). The three treated groups showed higher *Nrf2* and *HO-1* gene expression than in the model group (*p* < 0.05), as detailed in Table 5. *Nrf2* and *HO-1* expression levels in the TCM group were similar to those in the WM group, with no significant differences. However, the TCM-WM combination group showed significantly higher *Nrf2* and *HO-1* gene expression than both the TCM and WM groups (*p* < 0.05).

**Table 5:** Comparative analysis of Nrf2 and HO-1 gene copy numbers in skeletal muscles of five groups of mice

Group	*n*	Nrf2	HO-1
Control	8	1.000 ± 0.024	1.008 ± 0.020
COPD model	8	0.445 ± 0.138	0.795 ± 0.110
TCM	8	0.734 ± 0.066^*^	1.177 ± 0.145^*^
WM	8	0.662 ± 0.110^#^	1.262 ± 0.181^#^
TCM-WM combination	8	0.852 ± 0.071^@^	1.416 ± 0.156^@^
f value		42.03	25.21
P value		< 0.001	< 0.001

**Note**: For Nrf2 gene copy numbers, compared with the control group, *P* < 0.001 for all four model groups; compared with the model group, *P* < 0.05 for the other three groups; compared with the WM group (^#^), ^*^P = 0.543, ^@^*P* = 0.011

For HO-1 gene copy numbers, compared with the control group, *P* < 0.05 for all four model groups; compared with the model group, *P* < 0.001 for the other three groups; compared with the WM group (^#^), ^*^P = 0.215, ^@^*P* = 0.029

## Discussion

Sarcopenia affects up to one-third of patients with COPD, with severity worsening with age and advancing airflow limitation [10]. Current studies have identified several mechanisms through which COPD leads to muscle atrophy, including hypoxemia, hypercapnia, systemic inflammation, oxidative stress, and apoptosis [11]. The modified Jinshui Liujian decoction has shown effectiveness in managing both acute exacerbations and stable COPD phases. This formulation increases sputum production and aids phlegm expulsion, thus alleviating COPD symptoms through anti-inflammatory and antioxidant actions, inhibition of protease/antiprotease imbalance, airway remodeling, and reduction of mucus hypersecretion [12, 13]. This study used the modified Jinshui Liujian decoction combined with Bajitian pills, a formula derived from the Jingyue Quanshu and Taiping Shenghui Fang. The formula’s composition is carefully crafted: *Banxia* helps dry dampness and resolve phlegm; *Chenpi* regulates qi and invigorates the spleen; *Fuling* strengthens the spleen and promotes diuresis, complemented by *Gancao* to harmonize the formula. These herbs work synergistically to regulate qi, invigorate the spleen, dry dampness, and resolve phlegm. Additionally, *Danggui* and *Shudi* nourish yin and enrich blood, supporting lung and kidney functions. The principles of *Erchen Tang*—regulating qi, invigorating the spleen, and resolving phlegm—ensure smooth qi flow. *Xingren* and *Jiegeng* help disseminate lung qi, *Zhebeimu* clears heat and alleviates cough, *Ziyuan* and *Kuandonghua* resolve phlegm, while *Suzi* warms the lungs and supports kidney function. *Bajitian* and *Tusizi* invigorate the kidneys and fortify yang, *Wuweizi* consolidates the kidneys and restrains the lungs, *Shanyurou* supports the kidneys and stabilizes qi without aggravating fire, and *Buguzhi* and *Niuxi* enhance the liver and kidneys, fortifying muscles and bones. This formula provides cough suppression, phlegm resolution, spleen and lung reinforcement, kidney tonification, and muscle strengthening. It combines supplementation and purgation, addressing the lungs, spleen, and kidneys, offering a new therapeutic approach for sarcopenia linked to lung distension.

Network pharmacology is a crucial method for utilizing publicly available data platforms to explore the molecular targets and associated mechanisms through which TCM exerts therapeutic effects on various diseases [14]. In this study, network pharmacology analysis revealed that the modified Jinshui Liujian Decoction, in combination with Bajitian pills, impacts 74 core targets. Key targets, such as TP53, STAT3, AKT2, TNF, IL-6, and JUN, were identified, with TNF and IL-6 playing crucial roles in inflammatory responses, oxidative stress, and apoptotic pathways. GO enrichment analysis suggested that the decoction may affect metabolic processes, suppression of gene expression, apoptotic regulation, and inflammation, thereby modulating transcriptional activity and exerting anti-inflammatory effects. Additionally, KEGG enrichment analysis indicated that this TCM formula may reduce sarcopenia via pathways including cancer signaling, TNF signaling, and PI3K-Akt pathways. The network pharmacology results suggest that the modified Jinshui Liujian decoction and Bajitian pill combination, may safeguard against COPD-induced sarcopenia by reducing inflammation, mitigating oxidative stress, decreasing apoptosis, and modulating cancer-associated signaling pathways, thus providing a foundation for subsequent animal studies. Besides, the important position of Nrf2/HO-1 pathway as a functional nexus integrating multiple enriched pathways identified through KEGG analysis have been reported [15, 16], for example, regulating oxidative stress, inflammation and PI3K/Akt pathway. Therefore, Nrf2/HO-1 pathway has been selected as the studied pathway of applying TCM to treat COPD in further pharmacological experiment.

The mice displayed reduced vitality, dull fur, and decreased activity, while lung HE staining revealed alveolar wall rupture and inflammatory cell infiltration, confirming successful modeling [17]. The mice were randomized into three treatment groups: TCM, WM, and the combination of TCM and WM. The results showed that, compared to the COPD model group, the TCM group showed an increase in gastrocnemius muscle mass. HE staining revealed reduced muscle fiber atrophy and narrower interstitial spaces between fibers, suggesting that the modified Jinshui Liujian Decoction, combined with Bajitian Pills, protects skeletal muscle.

MDA, a byproduct of free radical damage, serves as an indirect marker of oxidative stress and free radical concentration in the body [18]. SOD is an antioxidative enzyme that mitigates oxidative stress and inhibits the activation of inflammatory mediators, playing a crucial role in the body’s defense mechanisms [19]. TNF-α enhances the synthesis of reactive oxygen species (ROS) and inflammatory mediators such as IL-6 and IL-1, activating inflammatory cells and amplifying the inflammatory response, while also influencing cellular apoptosis [20, 21]. Consequently, serum levels of MDA, SOD, and TNF-α are indicative of the degree of oxidative stress and inflammation within the body. The animal experiments demonstrated that, compared to the model group, the TCM group exhibited significantly lower MDA and TNF-α serum levels and higher SOD levels, indicating that the decoction effectively modulates systemic oxidative stress and inflammatory responses, which aligns well with the network pharmacology findings.

Nrf2 is a key transcription factor that regulates the expression of genes involved in antioxidant enzymes, anti-apoptotic proteins, and detoxification factors [22, 23]. HO-1, which is induced by Nrf2, plays a crucial role in anti-inflammatory and antioxidative stress responses by significantly suppressing pro-inflammatory cytokines [24]. A study by Cui et al. demonstrated that cigarette smoke extract inhibits the Nrf2/HO-1 signaling pathway in human airway smooth muscle cells, exacerbating airway inflammation, a critical mechanism in COPD pathogenesis [25]. Oxidative stress is a pivotal factor in muscle atrophy, as ROS attack unsaturated fatty acids in cell membranes, leading to the release of lipid peroxides and promoting apoptosis [26, 27]. Activation of the Nrf2/HO-1 pathway has been shown to counteract the harmful effects of ROS in skeletal muscle mitochondria, protecting muscle fibers and reducing myocyte apoptosis [28]. Furthermore, studies have indicated that Nrf2 deficiency accelerates muscle cell degradation, with rapid clearance of autophagy-related proteins in skeletal muscle and an excessive increase in autophagic activity, both contributing to muscle atrophy [7, 8]. Consequently, the Nrf2/HO-1 pathway presents a potential therapeutic target for treating COPD-induced muscle atrophy.

The aim of this study was to investigate the effect of the modified Jinshui Liujian decoction and Bajitian pill combination on the Nrf2/HO-1 pathway in skeletal muscle cells. The results showed a significant increase in HO-1 protein and gene expression compared to the model group, indicating that this formula protects skeletal muscle by modulating the Nrf2/HO-1 pathway and delaying muscle atrophy.

In this experiment, a comparison between the TCM group, WM group, and TCM-WM combination group revealed that the therapeutic outcomes of the individual TCM and WM groups were similar. However, the TCM-WM combination group showed more pronounced changes in serum MDA, TNF-α, and SOD levels, along with increased muscle mass, narrower fiber spacing, and higher expression of Nrf2 and HO-1 in the gastrocnemius muscle, compared to the individual TCM and WM groups. These differences were statistically significant, indicating that combination therapy is the most effective.

Integrative therapy combining TCM and WM is currently widely used in the clinical treatment of COPD. Xiong et al. reported that herbal medicine is a relatively safe treatment option that alleviates COPD symptoms and reduces the frequency of acute exacerbations [29]. Similarly, Xu et al. found that personalized TCM combined with WM treatment reduces mortality in hospitalized patients with COPD [29]. This study demonstrates that the modified Jinshui Liujian decoction and Bajitian pill combination, when used along with WM, significantly reduces systemic inflammation and oxidative stress, delays muscle atrophy, and prevents sarcopenia.

## Conclusion

In summary, this study provides evidence supporting the use of the modified Jinshui Liujian decoction and Bajitian pill combination for treating COPD-induced sarcopenia. The decoction inhibits muscle atrophy through various pathways and targets, particularly by regulating the Nrf2/HO-1 pathway. The combination of TCM and WM provides enhanced therapeutic efficacy for patients with COPD-induced sarcopenia.

## Electronic supplementary material

Below is the link to the electronic supplementary material.

Supplementary Material 1

## Data Availability

The datasets used or analysed during the current study are available from the corresponding author on reasonable request.

## Abbreviations

COPD: Chronic obstructive pulmonary disease
WM: Western medicine
TCM: Traditional Chinese medicine
Nrf2: Nuclear factor erythroid 2-related factor 2
PPI: Protein-protein interaction
GO: Gene ontology
KEGG: Kyoto encyclopedia of genes and genomes
LPS: Lipopolysaccharide
MDA: Malondialdehyde
SOD: Superoxide dismutase
TNF-α: Tumor necrosis factor-α
HO-1: Heme oxygenase-1
