# Supplementary figures and images for: Regulation of the Nrf2/HO-1 pathway in chronic obstructive pulmonary disease-induced muscle atrophy using Jinshui Liujian decoction and Bajitian pills: insights from network pharmacology and animal models
Source: Hereditas. 2025 Apr 21;162:66. doi: 10.1186/s41065-025-00432-5 (PMC12012937; doi:10.1186/s41065-025-00432-5)

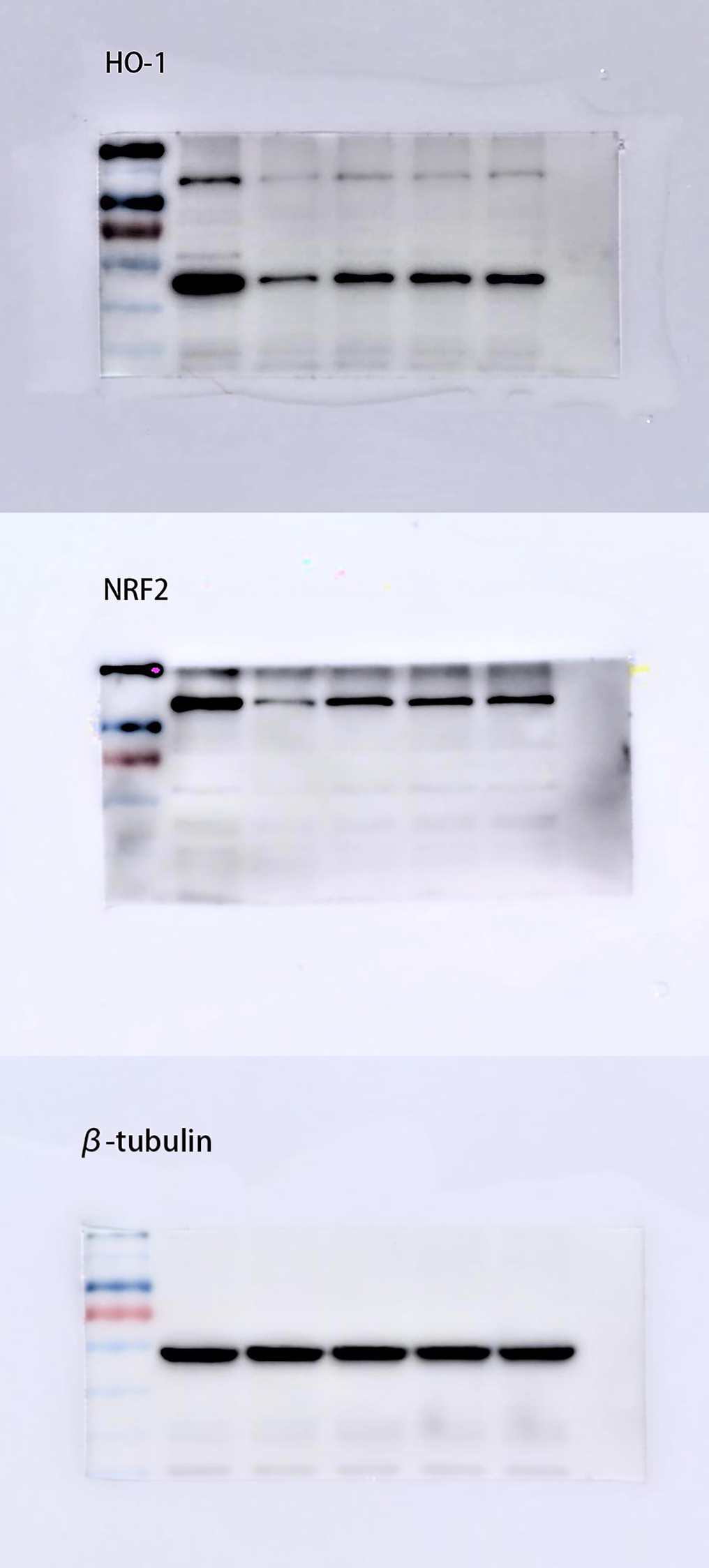

Supplement: Supplementary file 1 — Supplementary Material 1 [file 41065_2025_432_MOESM1_ESM.tif]
